# Sulfonamide Per- and Polyfluoroalkyl Substances Can Impact Microorganisms Used in Aromatic Hydrocarbon and Trichloroethene Bioremediation

**DOI:** 10.1021/acs.est.3c09715

**Published:** 2024-05-08

**Authors:** Emily K. Cook, Christopher I. Olivares, Edmund H. Antell, Katerina Tsou, Tae-Kyoung Kim, Amy Cuthbertson, Christopher P. Higgins, David L. Sedlak, Lisa Alvarez-Cohen

**Affiliations:** † Department of Civil and Environmental Engineering, University of California, Berkeley, California 94720, United States; ‡ Department of Civil and Environmental Engineering, University of California, Irvine, California 92697, United States; § Department of Civil & Environmental Engineering, Colorado School of Mines, Golden, Colorado 80401, United States

**Keywords:** PFAS, AFFF, groundwater bioremediation, microbial toxicity

## Abstract

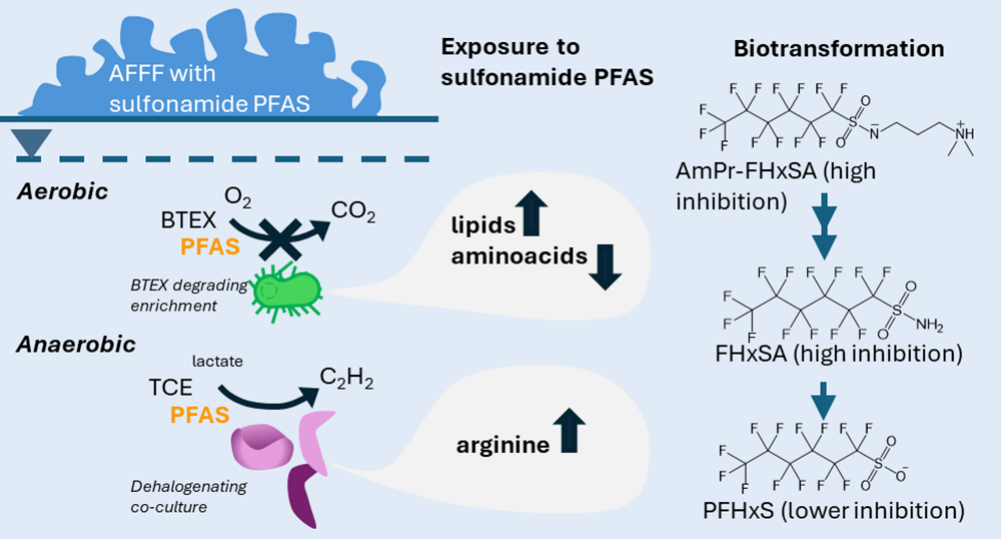

Per- and polyfluoroalkyl substances (PFASs) from aqueous film forming foams (AFFFs) can hinder bioremediation of co-contaminants such as trichloroethene (TCE) and benzene, toluene, ethylbenzene, and xylene (BTEX). Anaerobic dechlorination can require bioaugmentation of *Dehalococcoides*, and for BTEX, oxygen is often sparged to stimulate in situ aerobic biodegradation. We tested PFAS inhibition to TCE and BTEX bioremediation by exposing an anaerobic TCE-dechlorinating coculture, an aerobic BTEX-degrading enrichment culture, and an anaerobic toluene-degrading enrichment culture to *n*-dimethyl perfluorohexane sulfonamido amine (AmPr-FHxSA), perfluorohexane sulfonamide (FHxSA), perfluorohexanesulfonic acid (PFHxS), or nonfluorinated surfactant sodium dodecyl sulfate (SDS). The anaerobic TCE-dechlorinating coculture was resistant to individual PFAS exposures but was inhibited by >1000× diluted AFFF. FHxSA and AmPr-FHxSA inhibited the aerobic BTEX-degrading enrichment. The anaerobic toluene-degrading enrichment was not inhibited by AFFF or individual PFASs. Increases in amino acids in the anaerobic TCE-dechlorinating coculture compared to the control indicated stress response, whereas the BTEX culture exhibited lower concentrations of all amino acids upon exposure to most surfactants (both fluorinated and nonfluorinated) compared to the control. These data suggest the main mechanisms of microbial toxicity are related to interactions with cell membrane synthesis as well as protein stress signaling.

## Abstract 2

The charge and sulfonamide moiety of PFASs affect their microbial toxicity as PFASs induce protein biosynthesis and impact cell membranes.

## Introduction

Per- and polyfluoroalkyl substances (PFASs) are performance-enhancing substances in aqueous film-forming foams (AFFFs), added for their ability to drastically lower the surface tension of a solution, increasing an AFFF’s ability to quickly quench fuel and solvent fires.^1^ Historically, 3M’s electrochemical fluorination (ECF)-based AFFF formulations contained PFASs such as perfluorooctanesulfonate (PFOS), which is both bioaccumulative and toxic,^2,3^ as well perfluorohexanesulfonate (PFHxS) and *n*-dimethyl ammonio propyl perfluorohexane sulfonamide (AmPr-FHxSA).^4^ Recently, researchers have frequently detected PFHxS and AmPr-FHxSA in groundwater, as well as perfluorohexane sulfonamide (FHxSA), an aerobic biotransformation product of perfluorohexyl-based ECF precursors including AmPr-FHxSA.^5−8^ In one study, FHxSA was the dominant ECF-based sulfonamide present in groundwater samples.^9^ Several groups have reported biotransformation pathways and rates of ECF-based precursors,^5,8,10−12^ but we lack information on the inhibitory effects of ECF precursors and their transformation products on microorganisms that play key roles in bioremediation.

Few studies have examined PFAS effects on prokaryotes.^13−18^ When aerobic toluene-degrading *Rhodococcus jostii* strain RHA1 was exposed to a combination of 11 perfluoroalkyl acids (PFAAs) (10 mg/L each for a total PFAS exposure of 110 mg/L), toluene degradation was not impacted by PFASs, but the cells experienced enhanced aggregation as well as upregulation of stress-related genes.^19^ In another study, partitioning of PFAAs into bacterial cell membranes and model membranes increased with PFAS chain length.^20^ This effect was greater for perfluoroalkyl sulfonic acids (PFSAs) compared to perfluoroalkyl carboxylic acids (PFCAs) and higher for Gram-negative cells.^20^ PFAS disruption of cell membranes is important because an increase in membrane fluidity can disrupt cross-membrane proton gradients required for energy production via the electron transport chain. This group also investigated the effect of PFAAs on quorum sensing and bioluminescence of aerobic marine bacterium *Aliivibrio fischeri*.^15^ They observed that 50 mg/L of perfluorobutanesulfonic acid (PFBS) and, to a lesser extent, PFHxS increased metabolic rates of the bacterium, possibly due to proton leakage from compromised membrane integrity. PFOS concentrations of 50 mg/L decreased its metabolic rate due to acute toxicity.^15^ In contrast, anaerobic dechlorinating mixed cultures were inhibited by PFASs,^17,18^ although this was not observed with a commercial dechlorinating consortium.^16^ High concentrations (i.e., 16 and 32 mg/L) of the precursor fluorotelomer sulfonamido betaine (FtSaB) have been found to inhibit TCE degradation, whereas fluorotelomer thioether amido sulfonate (FtTAoS) (45 mg/L) did not. It was hypothesized that the positively charged betaine group in FtSaB was in part responsible for this difference^17^ as cationic amines are potent antibacterials.^21^

Although it is necessary to test the effect of individual PFASs to develop an understanding of the underlying mechanisms of toxicity, AFFF formulations contain multiple fluorinated and nonfluorinated surfactants and other potentially inhibitory chemicals such as glycol solvents and biocides.^22^ In a previous study, an anaerobic trichloroethene (TCE)-dechlorinating enrichment culture was exposed to fluorotelomer and ECF-based AFFFs.^17^ It was found that fermentable organics in AFFFs stimulated anaerobic dehalogenation, whereas cationic fluorotelomer PFASs inhibited it.^17^ Specifically, fluorotelomer-based AFFFs inhibited TCE dechlorination, whereas the ECF-based 3 M AFFF did not.^17^

These studies provide insight into how PFASs impact bacterial cells, but some data gaps remain. For example, six carbon PFASs have been understudied compared to their eight carbon congeners, yet their physicochemical properties make them interact with biological membranes differently.^20^ Further, the mechanisms driving inhibition are still unclear, particularly under different redox conditions. We sought to evaluate the potential toxicity of hexafluoroalkyl ECF-based PFASs, which are the most abundant precursors in ECF-based AFFF.^23,25^ We focused specifically on zwitterionic AmPr-FHxSA and its biotransformation products FHxSA and PFHxS, as well as a 3M AFFF containing high concentrations of AmPr-FHxSA and PFHxS. In particular, we wanted to determine if hexafluoroalkyl PFASs negatively impact microorganisms capable of degrading common PFAS source zone co-contaminants such as benzene, toluene, ethylbenzene, and xylene (BTEX) and TCE. We hypothesized that an aerobic enrichment would be more robust compared to anaerobic enrichments and that the AFFF would be more inhibitory to the cultures compared to the individual PFASs.

In this study, we exposed an aerobic BTEX-degrading enrichment culture, an anaerobic toluene-degrading enrichment (both seeded from AFFF-contaminated sites), and an anaerobic TCE-dechlorinating coculture to 1 and 10 μM (i.e., approximately 0.5 and 5 mg/L) individual ECF-based PFASs and dilutions of an ECF-based AFFF to determine possible inhibitory effects on these bacteria. We chose these concentrations to be representative of groundwater in AFFF-impacted source zones.^24,25^ We investigated the effects of electron donor/acceptor consumption, adenosine triphosphate (ATP) production, amino acid production, and community changes to determine how bioremediation processes may be affected by the presence of AFFF-derived PFASs.

## Materials and Methods

### Chemicals

PFAS analytical standards (including C4–C10 PFCAs, FHxSA, and AmPr-FHxSA) and mass labeled standards were purchased from Wellington Laboratories (Guelph, ON, Canada). BTEX compounds (>98%) were purchased from Sigma-Aldrich, and TCE was from Acros Organics (99.6%). All other chemicals used in media or analyses, including LC-grade water, methanol, acetonitrile, and isopropanol, were purchased from Sigma-Aldrich or Fisher Scientific at the highest purity available. The ECF-based AFFF used, a Cal Guardian 3M AFFF 3%, was donated by Prof. Jennifer Field at Oregon State University; PFAS concentrations for this AFFF are shown in our prior work.^8^

### Microcosms

#### Anaerobic TCE-Dechlorinating Coculture

**Figure 1:**
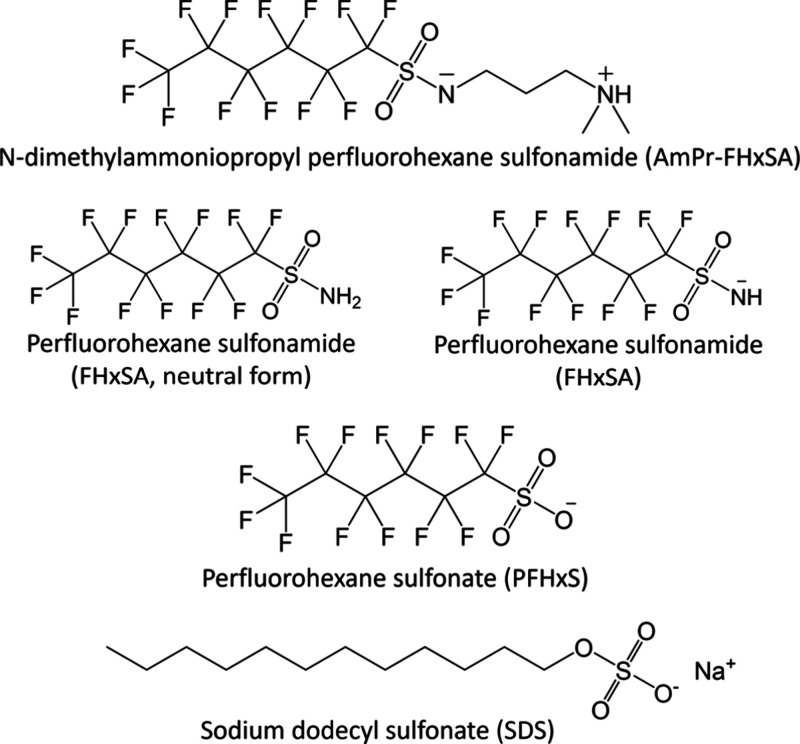
Surfactants tested in this study. All contained perfluorohexyl chains, except for the non-PFAS control, SDS. For FHxSA, both neutral and negatively charged forms are shown.

An anaerobic coculture of *Dehalococcoides mccartyi* strain 195 and *Desulfovibrio vulgaris* Hildenborough (Dhc195 and DvH, henceforth) was maintained (i.e., fed TCE and lactate regularly) in a 30 °C incubator until needed. For the inhibition tests, triplicates were exposed to PFHxS, FHxSA, AmPr-FHxSA, 3M AFFF, or the nonfluorinated surfactant sodium dodecyl sulfate (SDS) (Figure 1). Pure compounds were amended to a final concentration of approximately 1 or 10 μM, whereas AFFF was diluted (diluted 100, 1000, or 10,000×). The AFFF dilutions corresponded with approximately 300, 30, and 3 μM PFAS, respectively, with the concentrations mostly consisting of PFHxS, AmPr-FHxSA, and PFOS; for example, the 100× dilution was composed of 38 μM PFHxS, 65 μM AmPr-FHxSA, and 200 μM of PFOS. A triplicate set with no surfactant was included as a control. Glass serum bottles (160 mL) were amended with 94 mL of sterile BAV1 medium^8^ and 0.5 mL of a vitamin mixture as previously described,^26,27^ 0.5 mL of 1 M sodium lactate, 10 μL (14.6 mg) of neat TCE, and the respective surfactants with a headspace of N_2_/CO_2_ (80/20). Where aqueous surfactant stock solutions were possible (SDS, PFHxS, and diluted AFFF), the surfactant stock was added directly to reach the target concentration. For solvent-based surfactant stocks (FHxSA and AmPr-FHxSA), the required volume was first aseptically added to the empty and sterile bottles and dried with a slow stream of filtered (0.22 μm) N_2_. Then, the sterile medium components were added via a filtered needle syringe into the surfactant-containing bottles. The bottles were equilibrated for approximately 1 day before the addition of 5 mL of inoculum and experiment initiation. The bottles were incubated on their side in a 34 °C incubator without agitation (except during aqueous sampling) for the entirety of the experiment. The headspace was subsampled (100 μL) every 1–3 days for chlorinated solvent measurements as previously described,^27,28^ and aqueous samples were taken for ATP and metabolomics analyses at approximately the peak of substrate degradation (i.e., when microbial activity was expected to be the highest).

#### Aerobic BTEX-Degrading Enrichment Culture

An aerobic enrichment culture seeded from an AFFF-contaminated site^8^ has been maintained in our laboratory since early 2020 with regular feedings of BTEX, fresh medium, and oxygen. For the inhibition tests, 2.5 mL of this inoculum culture was added to 22 mL of AMS medium,^8^ 0.5 mL of a phosphate buffer, 10 μL of neat BTEX mixture (i.e., 2.5 μL of each compound), and the respective PFAS, as described above. The purpose of a lower volume in these batches was to maintain a high ratio of headspace to aqueous solution to ensure that oxygen was available to this aerobic culture. These bottles were kept on their sides in a 30 °C shaking incubator (150 rpm) for the experiment. Like the anaerobic experiments, these microcosms were first equilibrated without an inoculum for approximately a day for PFAS and BTEX equilibration in the aqueous phase. Headspace was sampled for BTEX components approximately every day, as previously described,^29^ and aqueous samples were taken at the peak of activity, as described above. This aerobic BTEX enrichment culture was additionally sampled for DNA extraction at the end of the 10 μM PFAS experiment for community comparison.

#### Anaerobic Toluene Enrichment Culture

The anaerobic toluene-degrading culture was started by adding AFFF-contaminated soil (from a U.S. Air Force Base) to bottles of anaerobic BAV1 medium, B12 vitamin mixture with a N_2_/CO_2_ 80/20 headspace with either excess nitrate or sulfate added as electron acceptors. For the inhibition tests, dilutions of the 3M AFFF (diluted 100, 1000, or 10,000×) or AmPr-FHxSA (1 or 10 μM) were added to 160 mL sealed glass serum bottles with 5 mL of inoculum, 94 mL of sterile BAV1 medium, 0.5 mL of B12 vitamins, and 10 μL of neat toluene.

### Metabolomics Analyses

Metabolomic extractions were performed based on Fiehn et al.^30^ Aqueous unfiltered (0.5 mL) and filtered (2 mL of BTEX aerobic enrichment and 5 mL of the anaerobic TCE-dechlorinating coculture) samples were taken near the end of the first cycle of substrate/electron acceptor degradation and frozen at −20 °C for up to 1 week until extraction.^31^ Samples were thawed over ice and mixed with cold 1:1 isopropanol/acetonitrile at a 1:3 ratio (e.g., 0.5 mL of aqueous sample, 1.5 mL of solvent mixture, kept at −20 °C before use), vortexed for 10 s, shaken for 5 min at 4 °C, and centrifuged at 12,800*g* for 2 min at 4 °C. About 90% of the supernatant was removed, and the remaining sample was dried and resuspended in 250 μL of 1:1 acetonitrile:water. This mixture was vortexed briefly and centrifuged for 2 min at 12,800*g*. Approximately 150 μL of the supernatant was transferred into a vial for LC–MS/MS analysis, and the remaining 100 μL was transferred to a 2 mL centrifuge tube for subsequent derivatization for GC–MS metabolomic analysis.

For the derivatization, the 100 μL mixture was dried and then supplemented with 10 μL of a 28 mg/mL methoxyamine hydrochloride solution in pyridine, 20 μL of pyridine, and 5 μL of d-myristic acid (0.75 mg/mL in hexane) and vortexed at 40 °C for 90 min. Then, 70 μL of *N*-methyl-*N*-(trimethylsilyl)trifluoroacetamide (MSTFA) was added, and samples were vortexed again at 40 °C for 50 min. This mixture was transferred to a vial for full-scan GC–MS analysis.

Derivatized samples were run on a GC–MS (Agilent 5977B) with a 1 μL injection volume. A DB5 capillary column (30 m x 250 μm i.d., 0.25 μm film thickness; J&W Scientific, Folson, CA) was used with a programmable temperature vaporizing injection (Gerstel CIS4 injector) in splitless mode. Constant flow (1.7 mL/min) was used; the MS was used in the electron impact mode, and we additionally ran full scans for each sample using *m*/*z* ratios 15–800. The inlet temperature was set at 125 °C, and the ion source and quadrupole were maintained at 250 and 200 °C, respectively. Raw data files were processed using MS-Dial 4.9^32^ for peak deconvolution and fatty acid methyl ester retention index alignment.^30^ Metabolite identification was based on scoring at 70% similarity based on the EI mass spectrum and retention index tolerance (3000). Log2 fold changes of treatment peak heights were compared against the control, and we chose two-tailed paired *t* tests (α = 0.05) to evaluate the statistical significance of mean peak height (*n* = 3) between the control and each of the PFAS or SDS treatments.

Additional underivatized extracts were used to evaluate amino acids using LC–MS/MS.^33,34^ The acetonitrile/water samples were run using a HILIC Plus column (4.6 × 100 mm, 3.5 μm). The samples were run at 0.4 mL/min using a mobile phase gradient (Table S2): organic phase (95% acetonitrile, 5% water, 0.1% formic acid, with 10 mM ammonium formate) and aqueous phase (0.1% formic acid and 10 mM ammonium formate). Amino acid standards were run to confirm the ion transitions and retention times. Similar to GC–MS metabolites, we used two-tailed paired *t* tests of Log2 fold changes using treatment/control peak areas.

### Additional Microbial Analyses

ATP was measured using an assay adapted from Hammes et al.^35^ as previously described in Kennedy et al.^36^ and Miller et al.^37^ Briefly, immediately after sample collection, filtered (0.22 μm) (for extracellular ATP concentrations) and unfiltered (for total ATP concentrations) microcosm samples were diluted 10–100× with Milli-Q water. Five hundred microliters of this dilution and a 50 μL aliquot of the ATP Reagent BacTiter-Glo (G8231, Promega Corporation, Madison, WI) were each incubated separately for 3 min at 38 °C; then, the reagent and sample were combined, incubated for an additional 20 s, and measured using a luminometer (GloMax 20/20 Single Tube Luminometer, Model No. E6080, Promega Corporation, Madison, WI). Sample concentrations were compared to a standard curve made with dilutions of an ATP stock (P1132; Promega Corp., Madison, WI).

At the end of the BTEX enrichment experiments with 10 μM of each PFAS, 4 mL slurry samples were centrifuged at 10,000*g* for 10 min, decanted, and extracted for DNA using a DNeasy PowerSoil Kit (Qiagen, Hilden, Germany) according to the manufacturer’s instructions and as previously described.^8^ Extracted DNA was stored at −80 °C and sent to Novogene Corporation Inc. (Sacramento, CA, USA) for bacterial 16S rRNA amplification and sequencing. The raw sequence reads (.fq format) have been deposited in the Dryad Digital Repository.^38^ Data processing was performed as previously described.^8^

## Results and Discussion

### Co-contaminant Degradation

Dhc195 and Dvh exist in a syntrophic relationship that results in more rapid and robust dechlorination of TCE to ethene, as previously described.^27^ Dhc195 uses chlorinated solvents as electron acceptors, transforming TCE to *cis*-dichloroethene (cis-DCE), vinyl chloride (VC), and finally ethene with the support of sulfate-reducing Dvh. When we exposed this coculture to 1 and 10 μM individual PFASs and SDS, there were no effects on the rate of dehalogenation of TCE to ethene compared to the no-surfactant controls (Figure S1).

**Figure 2:**
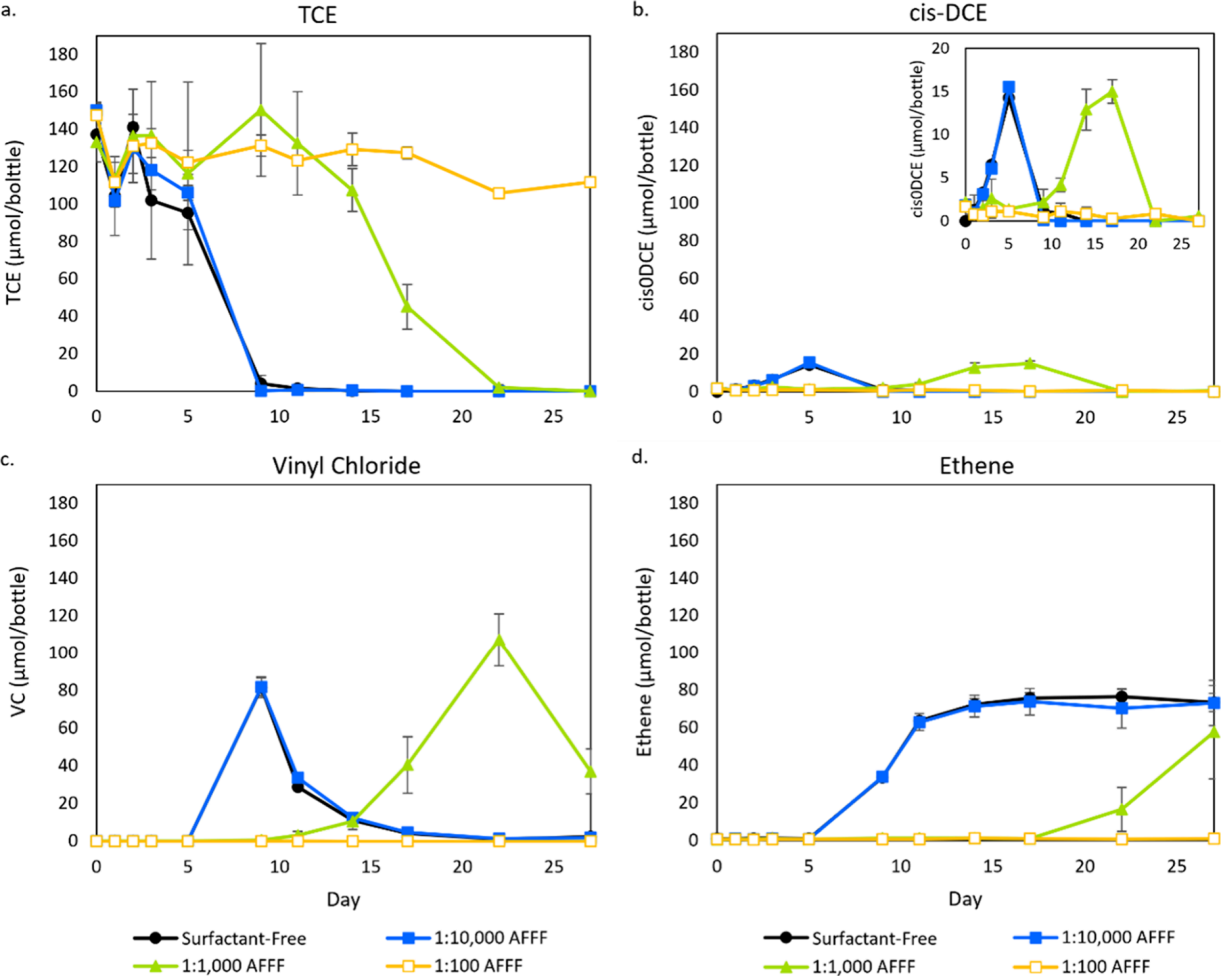
(a) TCE dechlorination and subsequent production of (b) *cis*-DCE, (c) VC, and (d) ethene by the anaerobic coculture. This culture was exposed to increasing dilutions of an ECF-based AFFF. Error bars represent the standard deviation of triplicate bottles.

We repeated this experiment with three dilutions of the AFFF: 100, 1000, and 10,000×, which correspond to approximately 300, 30, and 3 μM of PFAS, respectively, consisting of PFHxS, AmPr-FHxSA, and PFOS as described in Materials and Methods (Figure 2). For reference, 100× dilution corresponds to approximately one-third of the strength of the released AFFF based on a 3% application. The 10,000× diluted replicates performed similarly to the control, whereas the 1000× diluted set resulted in a slower though still complete degradation of TCE to ethene (Figure 2). In contrast, the microcosms that contained 100× diluted AFFF resulted in the complete inhibition of TCE dechlorination with no production of *cis*-DCE, VC, or ethene (Figure 2). Besides the identified PFASs in the AFFF, there are other AFFF components that could have contributed to inhibition but were not evaluated in this study. For example, Tsou et al. detected untargeted short and ultrashort (less than four perfluorinated carbons) precursors based on an improved total oxidizable precursor (TOP) assay on the AFFF used in this study,^23^ the potential toxicity of which is not understood.

**Figure 3:**
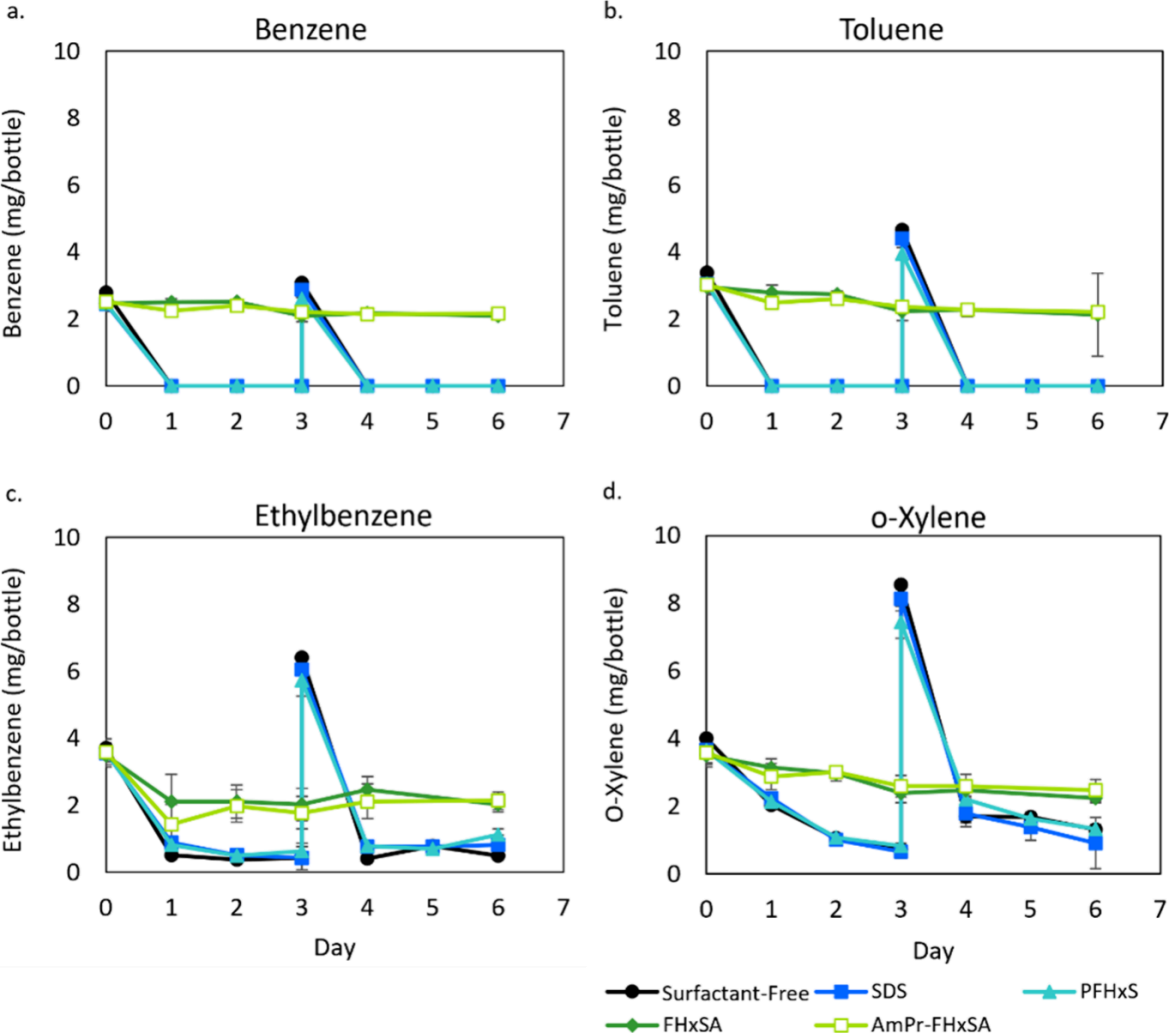
Aerobic degradation of (a) benzene, (b) toluene, (c) ethylbenzene, and (d) ortho-xylene by an AFFF-impacted enrichment culture in the presence of 10 μM of the individual PFAS, SDS, or the no-surfactant control. Error bars represent standard deviation of triplicate bottles. Additional BTEX was added on day 3 to all reactors that had already consumed most of their substrate; additional BTEX was not added to the AmPr-FHxSA and FHxSA cultures because they had not demonstrated BTEX degradation.

The aerobic BTEX-degrading enrichment culture, which uses BTEX as its carbon source and electron donor, did not exhibit any inhibitory effects when exposed to 1 μM PFAS and SDS (Figure S2). Exposure to either 10 μM FHxSA or AmPr-FHxSA, however, completely inhibited BTEX consumption compared to every other triplicate condition (Figure 3). Importantly, this effect was not observed for 10 μM PFHxS. Indeed, all triplicate sets, except for those exposed to AmPr-FHxSA or FHxSA, rapidly consumed the BTEX within a day and were consequently amended with BTEX components on day 3 while the experiment was continued to determine if the AmPr-FHxSA and FHxSA triplicate sets would demonstrate any substrate degradation.

When conducted with dilutions of AFFF (Figure S3), aerobic BTEX degradation in the 100 and 1,000× AFFF sets was slower compared to the control and 10,000× AFFF dilution. Because one of the organic components in AFFF with the highest concentrations (26–420 g/L) is diethylene glycol monobutyl ether (DGBE; commercially known as butyl carbitol),^39^ we tested its inhibition potential in additional toxicity tests but found no indication of inhibition of DGBE to aerobic BTEX biodegrading activity compared to the BTEX-only live control (Figure S4). Likewise, DGBE is fermentable and unlikely to pose inhibition to TCE-dechlorination.^17^ Other non-PFAS components in AFFF with biocide activity include hydrocarbon surfactants^40^ and the recently found benzotriazole corrosion inhibitors present in AFFF at 120–360 mg/L,^39^ which may also account for some inhibitory impact but were not tested individually in this study.

In isolation, PFHxS and SDS did not inhibit aerobic BTEX degradation, but FHxSA and AmPr-FHxSA did. AmPr-FHxSA is a zwitterion at neutral pH with a terminal cationic amine (calculated p*K*_a__1_ 3.57, p*K*_a__2_ 9.21),^41^ whereas most of FHxSA is in anionic form (calculated p*K*_a_ 6.3),^42^ suggesting that the majority of this PFAS is in anionic form at neutral pH. PFAAs can disrupt bacterial cell membranes,^15,19^ but the concentrations studied in this work did not appear to be sufficient to inhibit aerobic BTEX degradation. The biocidal activity of nonfluorinated surfactants is based on their ability to disrupt negatively charged microbial cell membranes,^43^ but the concentrations of the negatively charged SDS spiked in the experiments also had negligible effects on degradation. The effects of sulfonamide PFASs on membrane integrity have not been reported, to the best of our knowledge. It is possible that FHxSA could permeate cell membranes more effectively than its sulfonate analog, PFHxS, as reported for their C8 analogs.^44^ In addition, specific toxicity mechanisms may explain the increased level of inhibition of sulfonamides. Sulfonamides are known to limit bacterial growth because they interfere with folate biosynthesis.^45^ The zwitterion AmPr-FHxSA may have interfered with the membrane and membrane-bound proteins, such as ATP synthase, as described below. Surfactants with cationic amine functional groups, including polyfluoroalkyl precursors with cationic quaternary amine groups, have been shown to be biocidal as well as highly sorptive, contributing to their long-term stability and persistence.^5,11^

We also dosed an anaerobic toluene-degrading culture under nitrate- and sulfate-reducing conditions with the three AFFF dilutions and AmPr-FHxSA. Although this culture was enriched from the same soil as the aerobic BTEX-degrading enrichment culture, neither AmPr-FHxSA (1 or 10 μM) nor any of the three AFFF dilutions inhibited toluene degradation in these anaerobic cultures (Figures S5 and S6).

Finally, it should be noted that the highest concentrations of PFASs and AFFF dilutions tested impacted the solubility of the volatile co-contaminants, especially chlorinated solvents. This effect was evident under conditions with high PFAS concentrations and likely caused higher error bars in TCE (Figure 2 and Figure S1, day 0 in Figure S1e) as well as for BTEX (Figure S3). Many surfactants, not just PFASs, can affect the solubility of co-contaminants,^46,47^ demonstrating that at sites with volatile organics coexisting with AFFF-derived PFASs, solubility and detection may be impacted.

### Aerobic BTEX-Degrading Enrichment Community Composition

**Figure 4:**
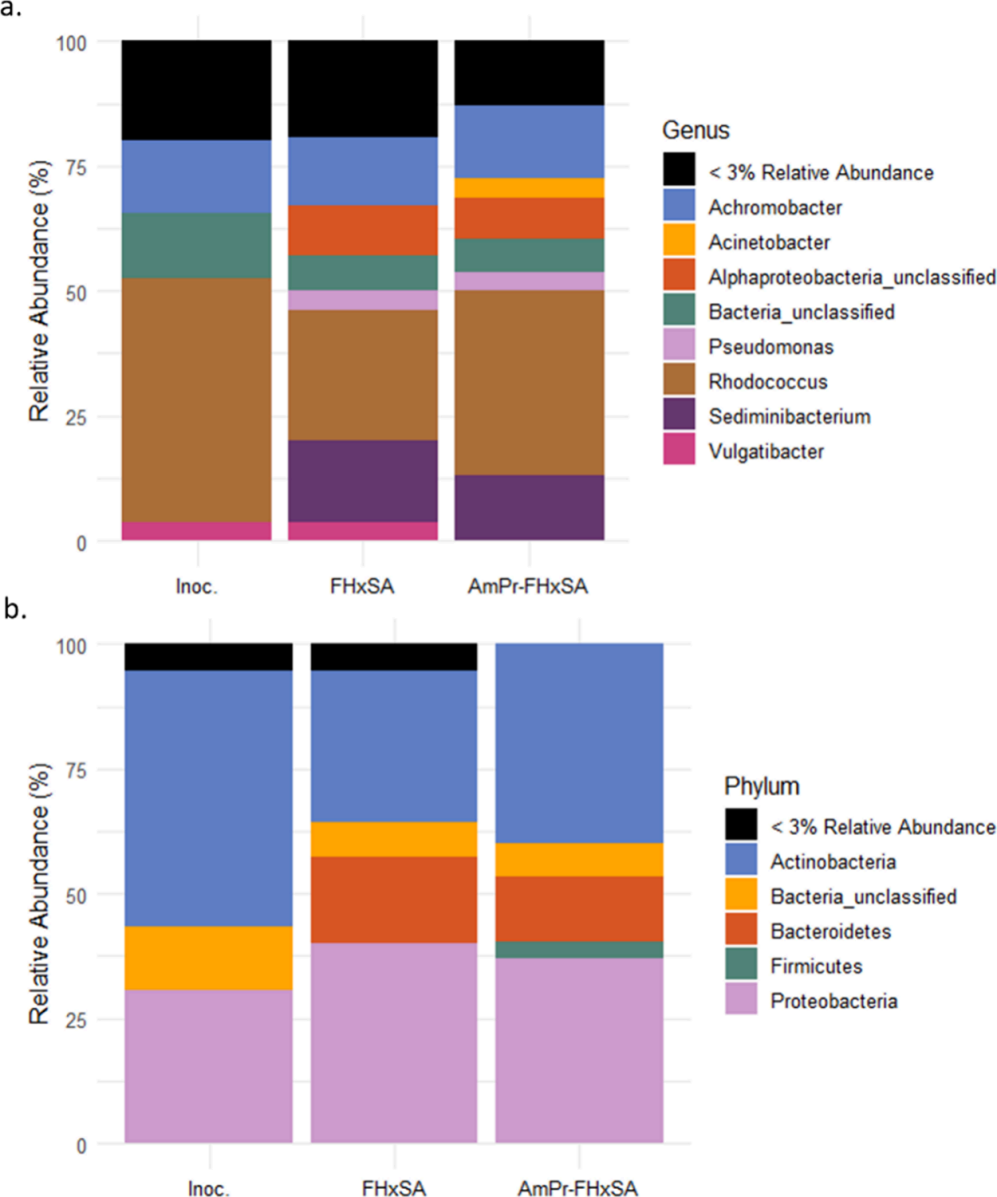
(a) Genera and (b) phyla of the inoculum, 10 μM FHxSA exposed cultures, and 10 μM AmPr-FHxSA exposed cultures in the aerobic BTEX-degrading enrichment culture.

Gram-negative bacteria seemed to be robust in exposures to 10 μM FHxSA and AmPr-FHxSA. On the genus level, the inoculum was dominated by two known BTEX degraders: *Rhodococcus* and *Achromobacter* (Figure 4a),^48,49^ as previously reported.^8^ PFAS exposures decreased the relative abundance of *Rhodococcus* (Gram-positive), but *Achromobacter* (Gram-negative) seemed unaffected based on its consistent relative abundance across PFAS cultures and the controls. Other Gram-negative bacterial species had detectable abundances in the PFAS-spiked cultures but not in the control. Cultures exposed to FHxSA and AmPr-FHxSA contained *Sediminibacterium,* unclassified Alphaproteobacteria, and *Pseudomonas*. The *Sediminibacterium* genus only has seven reported species,^50^ all of which are also Gram-negative heterotrophic aerobes. Alpha- and Gammaproteobacteria, all Gram-negative, were reported to increase in activated sludge exposed to perfluorooctanoic acid and PFOS.^51^ The inoculum and FHxSA culture shared a small abundance of *Vulgatibacter*. The literature on the *Vulgatibacer* genus, detected in the inoculum and in the FHxSA cultures, is sparse: this genus contains Gram-negative obligate heterotrophic aerobes.^52^ The AmPr-FHxSA exposed cultures were unique in containing *Acinetobacter*, Gram-negative bacteria known to be resistant to antibiotics.^53^*Acinetobacter* have been shown to be enriched using petroleum hydrocarbons as their carbon source and electron donor.^54,55^ Our finding that the most abundant genera in the PFAS-exposed cultures are Gram-negative bacteria is consistent with findings in other microbial systems exposed to PFASs.^51^ There are conflicting reports on higher adsorption capacity of anionic PFASs onto Gram-positive^56^ or Gram-negative^20,57^ bacterium models. If sorption to the cell membrane impacts cell function and decreases the abundance of bacteria, our findings suggest that the robustness of Gram-negative bacteria extends to perfluoroalkyl sulfonamides such as AmPr-FHxSA and FHxSA.

These findings are also detectable at the phylum level, with Actinobacteria and Proteobacteria as the most abundant detected phyla (Figure 4b). Exposures to FHxSA and AmPr-FHxSA increased the relative abundance of Proteobacteria and Bacteriodetes, Gram-negative bacterial genera. AmPr-FHxSA also had a detectable percentage of Firmicutes, which was undetected in the inoculum and in FHxSA exposures. Regarding cultures exposed to PFHxS and SDS, the DNA extraction and amplification efficiency was not acceptable and thus not reported.

### ATP Production

Some compounds, including surfactants like PFASs, can interfere with the generation of ATP.^58,59^ Specifically, surfactants can interact with cell membranes, causing a reduction in proton motive force and resulting ATP synthesis.^59^ Alternatively, surfactants can inactivate or extract membrane-bound proteins, such as ATPase.^60^ In the TCE dehalogenation tests, 1 and 10 μM surfactant additions (i.e., SDS, PFHxS, FHxSA, and AmPr-FHxSA) did not impact anaerobic dehalogenation (Figure S1) or ATP production (Figure S7). However, the AFFF-exposed treatments resulted in decreased dehalogenation (Figure 2) and decreased ATP production (Figure S8). ATP production in the anaerobic TCE-dechlorinating cocultures decreased approximately 77 and 82% in cultures exposed to 1000 and 100× diluted AFFF compared to the control on day 16 (Figure S8). The genus *Dehalococcoides* has a unique cell wall: instead of peptidoglycan, the cell wall structure resembles the S-layer protein subunit walls that are typical of archaea.^61^ These S-layer type proteins could be the main contributor to the coculture’s resistance to PFASs, especially when the coculture was exposed to compounds individually. Indeed, it has been shown that *Dehalococcoides* species can be resistant to antibiotics that target peptidoglycan biosynthesis.^62^

**Figure 5:**
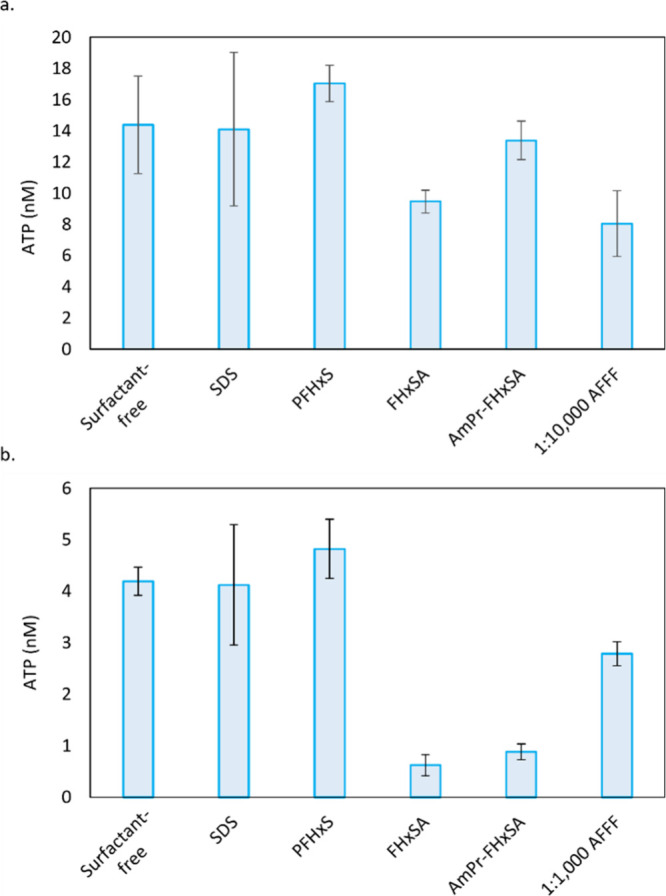
ATP concentrations in nM from the aerobic BTEX-degrading microcosms exposed to (a) 1 μm surfactants or 10,000× diluted AFFF versus (b) 10 μm surfactants or 1000× diluted AFFF. Error bars represent standard deviation of triplicate bottles.

Aerobic BTEX-degrading enrichment cultures exposed to 1 μM SDS and AmPr-FHxSA had similar ATP concentrations compared to the control (Figure 5). ATP production decreased approximately 34% in the cultures exposed to 1 μM FHxSA, although this culture showed no inhibition of BTEX consumption (Figure S2). The cultures exposed to 1 μM PFHxS did not have statistically significant differences in ATP production compared to the controls (paired *t* test, Figure 5). For the higher concentrations of PFASs, the addition of 10 μM SDS or PFHxS did not decrease the ATP concentrations (Figure 5). In the cultures exposed to 10 μM of FHxSA and AmPr-FHxSA, ATP production decreased by approximately 86% (FHxSA) and approximately 79% (AmPr-FHxSA) compared to the surfactant-free control. Both of these cultures had decreased BTEX degradation (Figure 3). We hypothesize that FHxSA was penetrating cell membranes in the aerobic BTEX culture, wheresa zwitterionic AmPr-FHxSA was associated with charged amine residues in membrane-bound proteins, inhibiting proper function. The cultures exposed to 10 μM PFHxS, similarly to the 1 μM PFHxS set, had increases in ATP production compared to those of the controls (Figure 5). It has been reported that at low concentrations (≤10 μM), SDS can stimulate ATPase activity before drastically reducing it above 20 μM.^60^ Although, in these experiments, the SDS conditions had ATP concentrations similar to the control, this finding in the literature^60^ could potentially explain the increase in ATP in the PFHxS cultures. Alternatively, this increase in ATP in the presence of PFHxS could be a result of differences in the microbial communities.

It has been shown that PFAS interference with cell membranes as well as proteins is chain length and headgroup dependent.^44,63,64^ For example, the types of amino acids near open binding sites can interact with different PFASs depending on the headgroup charge.^63^ Shen et al.^64^ showed that PFASs can enter a cell lipid bilayer by first switching orientation so that the fluorinated tail faces the membrane; this flip allows the PFAS to overcome the energy barrier associated with entry to the membrane. PFSAs penetrate the membrane bilayer better than PFCAs.^64^ In another study, the eight-carbon sulfonamide perfluorooctane sulfonamide (FOSA) incorporated twice as much into a phospholipid bilayer than PFOS.^44^ With increasing concentrations of PFASs, lipophobic PFASs in the lipid bilayer can result in lipid removal from the bilayer, which can cause a disordered membrane structure.^44^

Although the 1000× AFFF dilution in this experimental set (Figure 3) did not result in BTEX inhibition, the ATP concentrations in these cultures were approximately 66% of the control cultures (Figure 5). It is possible that because aerobic microorganisms produce more ATP than anaerobic ones,^65^ the 1 μM FHxSA set and the AFFF conditions simply reduced the cultures’ aerobic respiration ATP-production efficiency rather than completely inhibiting the cell’s ability to respire by inactivating proteins or destroying the cell membrane.

### Metabolites in Individual PFAS Exposures

**Figure 6:**
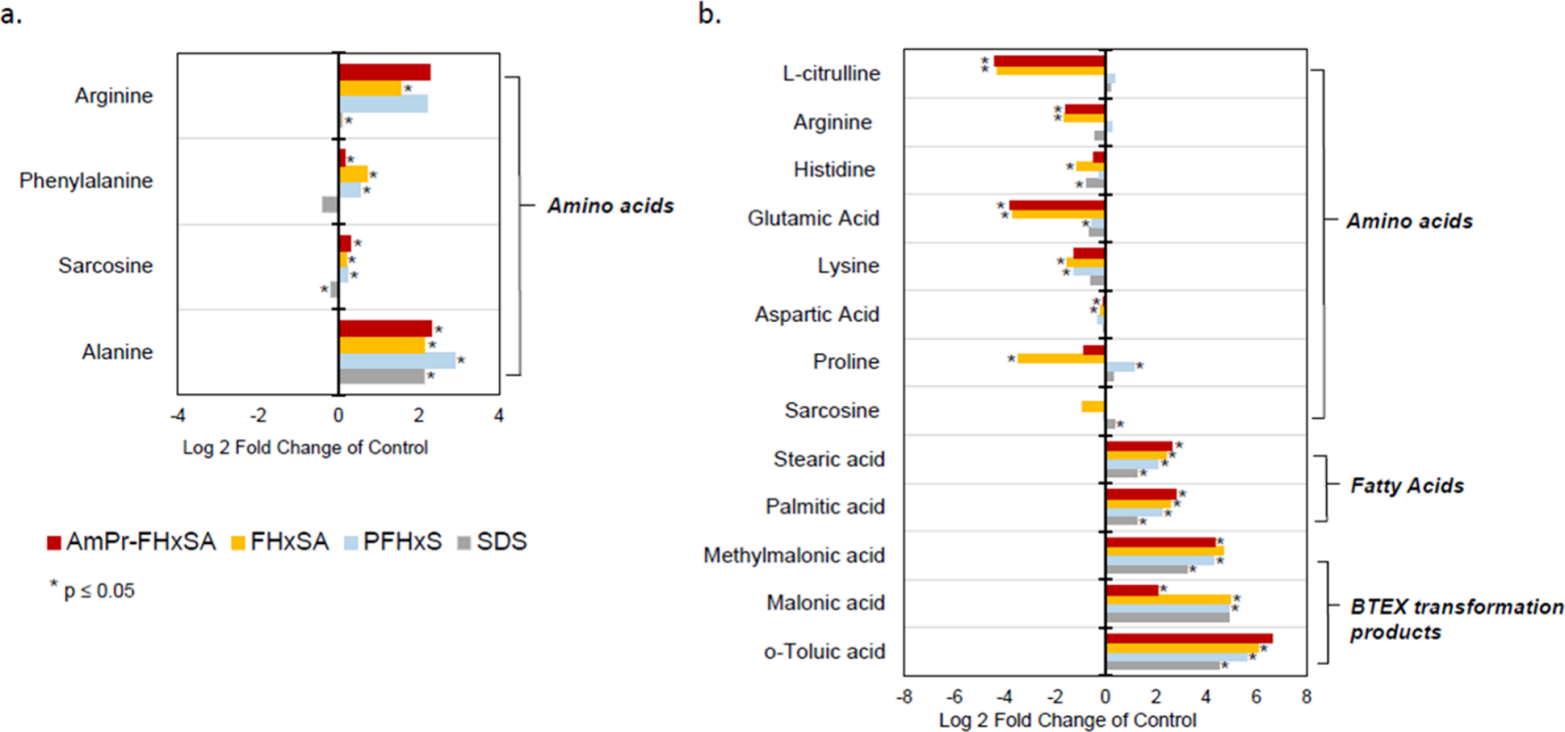
Log2 fold changes of total (intra- and extracellular) metabolite abundance detected in the (a) anaerobic TCE-dechlorinating coculture and (b) aerobic BTEX enrichment cultures exposed to AmPr-FHxSA. Asterisks denote fold changes with *p* ≤ 0.05.

**Figure 7:**
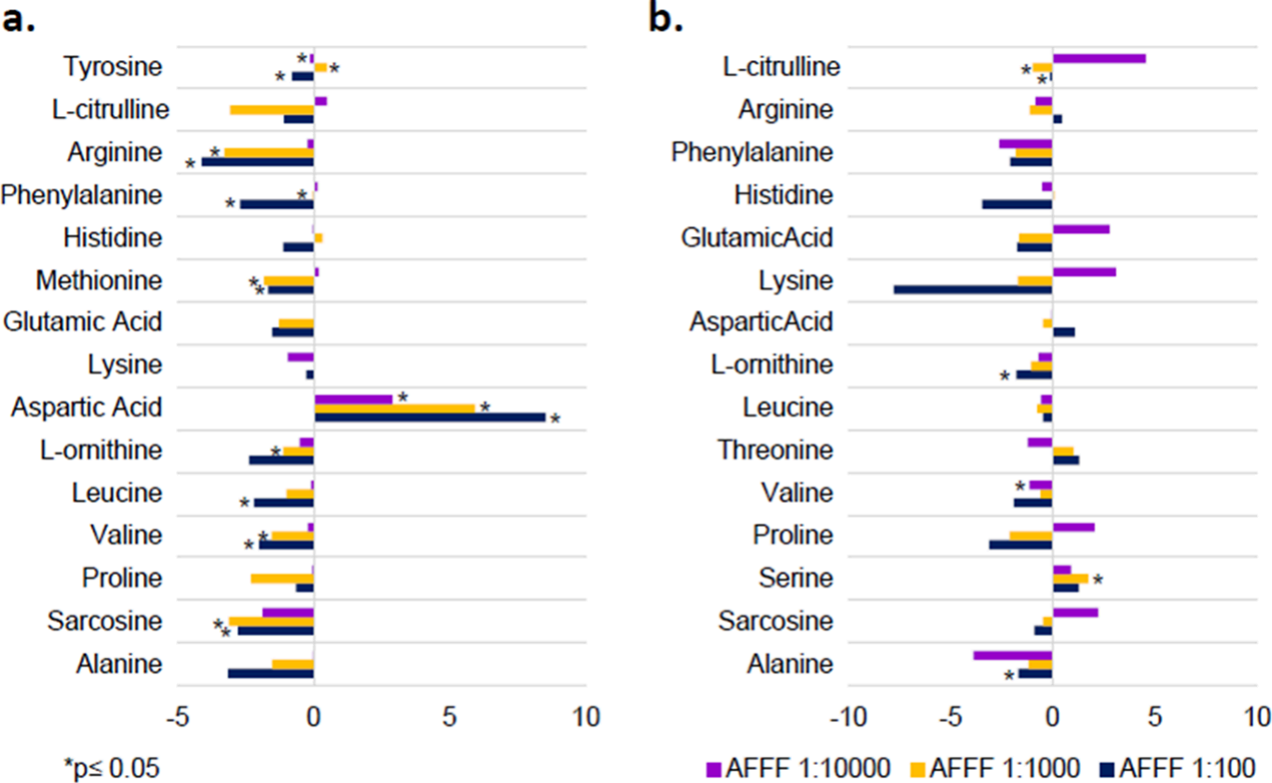
Metabolite Log2 fold changes with respect to AFFF-free controls measured in the (a) anaerobic TCE-dechlorinating coculture and (b) aerobic BTEX-degrading enrichment culture with the three dilutions of AFFF (1:10,000 the most diluted, AFFF 1:100 is the most concentrated)

We measured fold changes in metabolite abundances to study inhibition mechanisms of TCE and BTEX degradation (Figures 6 and 7) pathways upon exposure of AmPr-FHxSA, its transformation products, AFFF, and the fluorine-free surfactant SDS. Exposures of PFASs and SDS increased amino acid concentrations in the anaerobic TCE-dechlorinating coculture (Figure 6a). Two amino acids, arginine and phenylalanine, increased only in the PFAS-exposed treatments. Arginine and alanine had the greatest fold changes exposed to the PFAS compounds, but alanine also increased in the nonfluorinated SDS treatment. Arginine biosynthesis was also upregulated in *Dehalococcoides* during arsenic exposures.^27^ Both of these findings suggest that arginine may be a biomarker of xenobiotic stress in chlorinated solvent dehalogenation.

Unlike the anaerobic TCE-dechlorinating coculture, the aerobic BTEX-degrading enrichment culture had negative fold changes across all amino acids for individual PFASs and SDS (Figure 6b), with l-citrulline, glutamic acid, proline, and arginine with the largest concentration decreases when exposed to AmPr-FHxSA and FHxSA. Based on the KEGG *Rhodococcus* sp. pathways, glutamate is used in the synthesis of arginine. The aerobic BTEX-degrading enrichment culture had increases of unsaturated fatty acids, stearic acid, and palmitic acid (Figure 6b), but this effect was not specific to PFASs. These increases of fatty acids may be due to cell morphology changes during stress or cell membrane integrity losses.^13,15,66^ PFAAs have been reported to upregulate stress genes and stimulate the formation of extracellular polymeric substances in *Rhodococcus jostii*.^19^ Finally, we detected transformation intermediates of BTEX biodegradation: *o*-toluic (oxidation product of *o*-xylene), malonic, and methylmalonic acids (formed from ring cleavage of BTEX compounds). The increases in these transformation products signify incomplete biodegradation with respect to the surfactant-free control.

Taken together, our exposures of individual PFASs show that AmPr-FHxSA and its intermediate FHxSA negatively impacted aerobic BTEX biodegradation. Conversely, the anaerobic TCE-dechlorinating coculture was more resistant to inhibition from PFAS and AFFF exposures. The main mechanisms of toxicity in these microbial systems point to interactions with cell membrane synthesis as well as protein stress signaling stress. Considering individual PFASs, FHxSA and the zwitterionic AmPr-FHxSA showed higher potential for microbial inhibition compared with their anionic PFSA counterpart.

### Metabolites in AFFF Exposures

We also evaluated metabolite fold changes based on increasing dilutions of AFFF in both microbiological systems (Figure 7). An AFFF dilution-dependent fold change decrease of many of the metabolites can be seen in the aerobic BTEX-degrading and the anaerobic TCE-dechlorinating systems, suggesting a dose–response behavior in the metabolite abundances. In the anaerobic TCE-dechlorinating coculture, we observed concentration differences in amino acids involved in cellular signaling of stress and cell membrane biosynthesis compared to the non-AFFF control. Aspartic acid, used to synthesize lysine and peptidoglycan, had the highest Log2 fold change as AFFF concentrations increased (Figure 7a). Large increases in aspartic acid concentrations and the small, decreasing concentrations in lysine with increasing AFFF concentrations point to negative interactions of AFFF with peptidoglycan synthesis. Contrary to the metabolic profile of individual PFAS exposures (Figure 6a), most of the amino acids in the AFFF exposures had concentration decreases or negative fold changes, especially for arginine and sarcosine. The increasing doses of AFFF 1:1000 (∼30 μM PFASs) and 1:100 (∼300 μM PFASs) had slower dehalogenation activity (Figure 2), which corresponds with these decreases in arginine concentrations. The AFFF concentrations had a higher sum of total PFASs compared with the individual PFAS exposures, so the metabolite responses may be due to the cumulative toxicity effect or synergistic effects. As mentioned before, benzotriazole anticorrosives are also components in AFFF that may be responsible for the inhibition in AFFF dilutions.

The fold change in metabolite concentrations relative to the control in the BTEX enrichment had slight increases for the most diluted AFFF (1:10,000) but had negative fold changes for the more concentrated AFFF doses (Figure 7b). Lysine was the metabolite with the greatest negative fold change shift followed by glutamic acid, l-citrulline, proline, and sarcosine. Lysine is synthesized by several biosynthetic pathways in Gram-negative and Gram-positive bacteria, suggesting its metabolic importance for bacterial survival.^67^ Taken together, our AFFF exposures resulted in concentration-dependent decreases in metabolites, impacting primarily amino acid pathways related to stress and cell membrane biosynthesis.

## Implications

We demonstrated that a zwitterionic PFAS found in AFFF and its sulfonamide transformation product FHxSA resulted in increased microbial inhibition potential compared to their anionic PFSA analogue PFHxS at 1–10 μM individual exposures. Our findings show that PFASs can induce changes in metabolite abundance, especially lipids and some amino acids. In addition, AFFF 1:1000 dilutions (corresponding to 1/33 of the 3% field application or 30 μM total PFASs) inhibited co-contaminant bioremediation. Our findings are relevant for remediation of AFFF-impacted sites and suggest that reaeration of source zones with AFFFs containing AmPr-FHxSA may have reduced efficiency due to microbial inhibition to stimulated aromatic hydrocarbon degrading microorganisms. For sites where sulfonamide intermediates are commonly detected, such as FHxSA and FOSA, the incomplete biotransformation might be due to microbial inhibition caused by these sulfonamides. In contrast with BTEX biostimulation, bioaugmentation for anaerobic chlorinated solvent remediation might be uninhibited by the presence of PFASs. Although our study identified metabolite responses of PFAS stress to anaerobic TCE dechlorination and aerobic BTEX biodegradation, more mechanistic studies are needed to evaluate how these microbial metabolic processes are impacted by PFASs (e.g., mass labeled flux analyses of key metabolites, such as lipids and amino acids).

## Supporting Information Available

Aerobic BTEX and anaerobic BTEX, TCE degradation timelines, LC–MS/MS and GC–MS metabolite data, LC–MS/MS analysis additional details (PDF)
BTEX enrichment LC–MS metabolite data (XLSX)
BTEX enrichment GC–MS metabolite data (XLSX)
