# Supplementary material for: Sulfonamide Per- and Polyfluoroalkyl Substances Can Impact Microorganisms Used in Aromatic Hydrocarbon and Trichloroethene Bioremediation
Source: Environ Sci Technol. 2024 May 8;58(20):8792–802. doi: 10.1021/acs.est.3c09715 (PMC11112735; doi:10.1021/acs.est.3c09715)
Supplement: Supplementary file 1 — es3c09715_si_001.pdf [file es3c09715_si_001.pdf]

Sulfonamide per- and polyfluoroalkyl substances can impact microorganisms used in aromatic hydrocarbon and trichloroethene bioremediation

Supporting Information

Emily K. Cook[a], Christopher I. Olivares[a,b,*], Edmund H. Antell[a], Katerina Tsou[a], Tae-Kyoung Kim[a], Amy Cuthbertson[a], Christopher P. Higgins[c], David L. Sedlak[a], Lisa Alvarez-Cohen[a]

[a] Department of Civil and Environmental Engineering, University of California, Berkeley, CA, 94720, United States
[b] Department of Civil and Environmental Engineering, University of California, Irvine, CA, 92697, United States
[c] Department of Civil & Environmental Engineering, Colorado School of Mines, Golden, Colorado, 80401, United States
*Corresponding author: chris.olivares@uci.edu

Table of Contents

12 figures, 2 tables.

Figure S1 - Anaerobic TCE dehalogenation 1,10 µM exposures pg. S2

Figure S2 - Aerobic BTEX degradation, 1 µM exposures S3

Figure S3 – Aerobic BTEX degradation, AFFF exposures S4

Figure S4 – Aerobic BTEX degradation, AFFF vs. DGBE exposures S5

Figure S5 – Denitrifying toluene degradation, AFFF and Ampr-FHxSA exposures S6

Figure S6 – Sulfate-reducing toluene degradation, AFFF and Ampr-FHxSA exposures S7

Figure S7 – ATP for TCE co-culture, 10 uM exposures S8

Figure S8 – ATP for TCE co-culture, AFFF S9

Figure S9 – Heatmap metabolites TCE dehalogenation 1, 10 µM exposures S10

Figure S10 – Heatmap metabolites BTEX degradation, 1 µM exposures S11

Figure S11 – Heatmap metabolites BTEX degradation, 10 µM exposures S12

Table S1 – LCMS eluent gradient S13

Table S2 – LCMS metabolites details S14

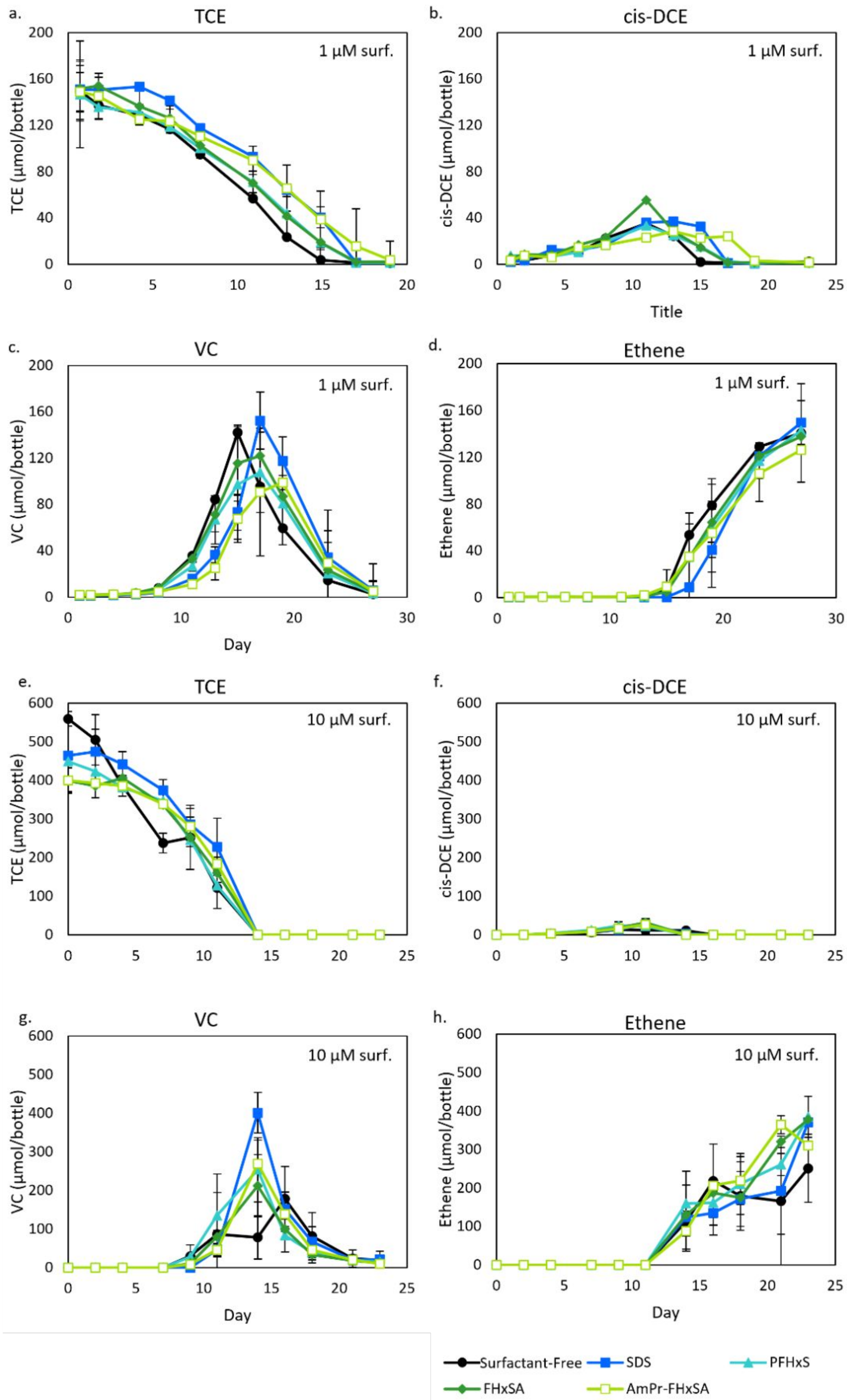

Figure S1. TCE (a,e), DCE (b,f), and VC (c,g) dehalogenation and ethene production (d,h) with the anaerobic culture exposed to 1 µM of surfactants (a-d) or 10 µM surfactants (e-g). Error bars represent standard deviation of triplicate cultures.

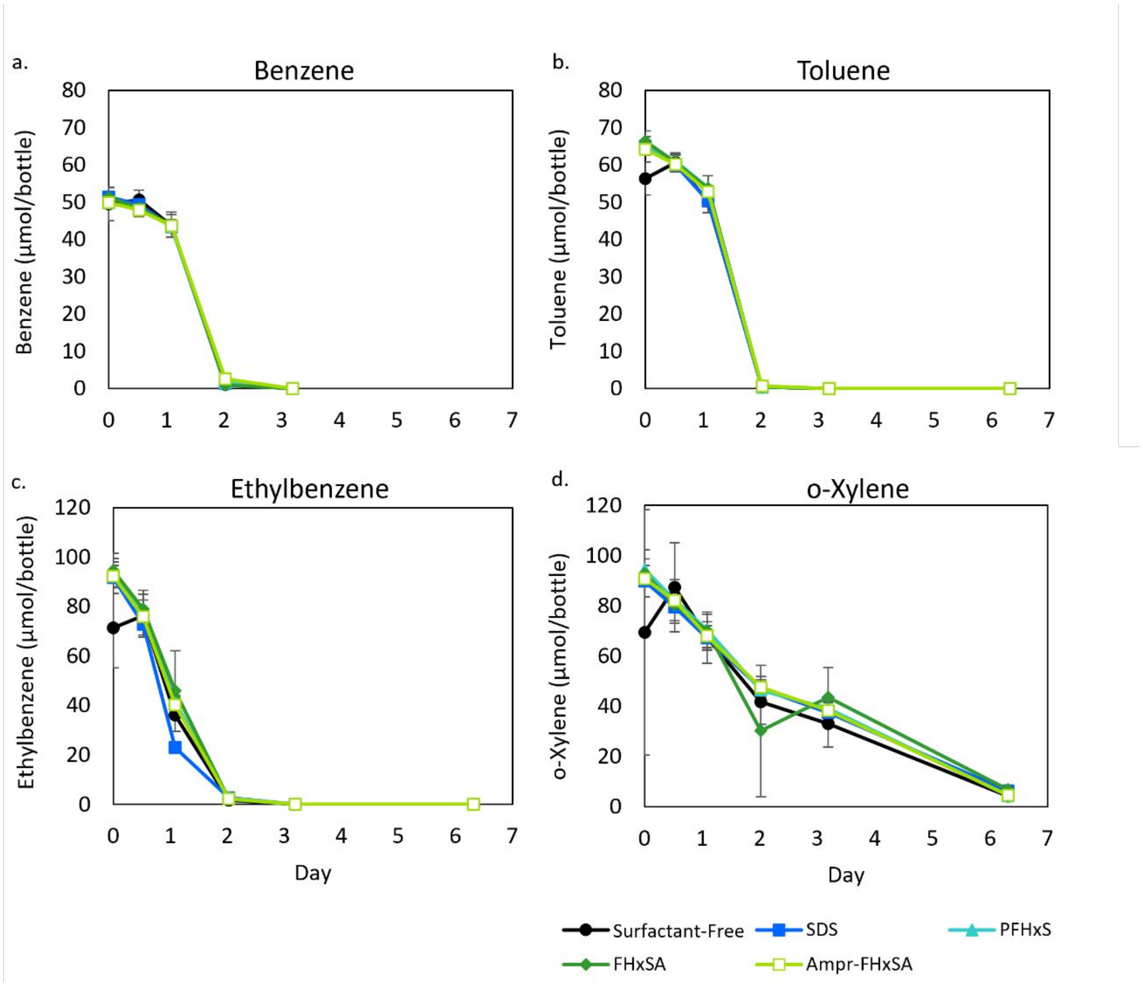

Figure S2. Aerobic BTEX degrading culture consuming a) benzene, b) toluene, c) ethylbenzene, and d) ortho-xylene in the presence of 1 µM surfactants. Error bars represent standard deviation of triplicate cultures.

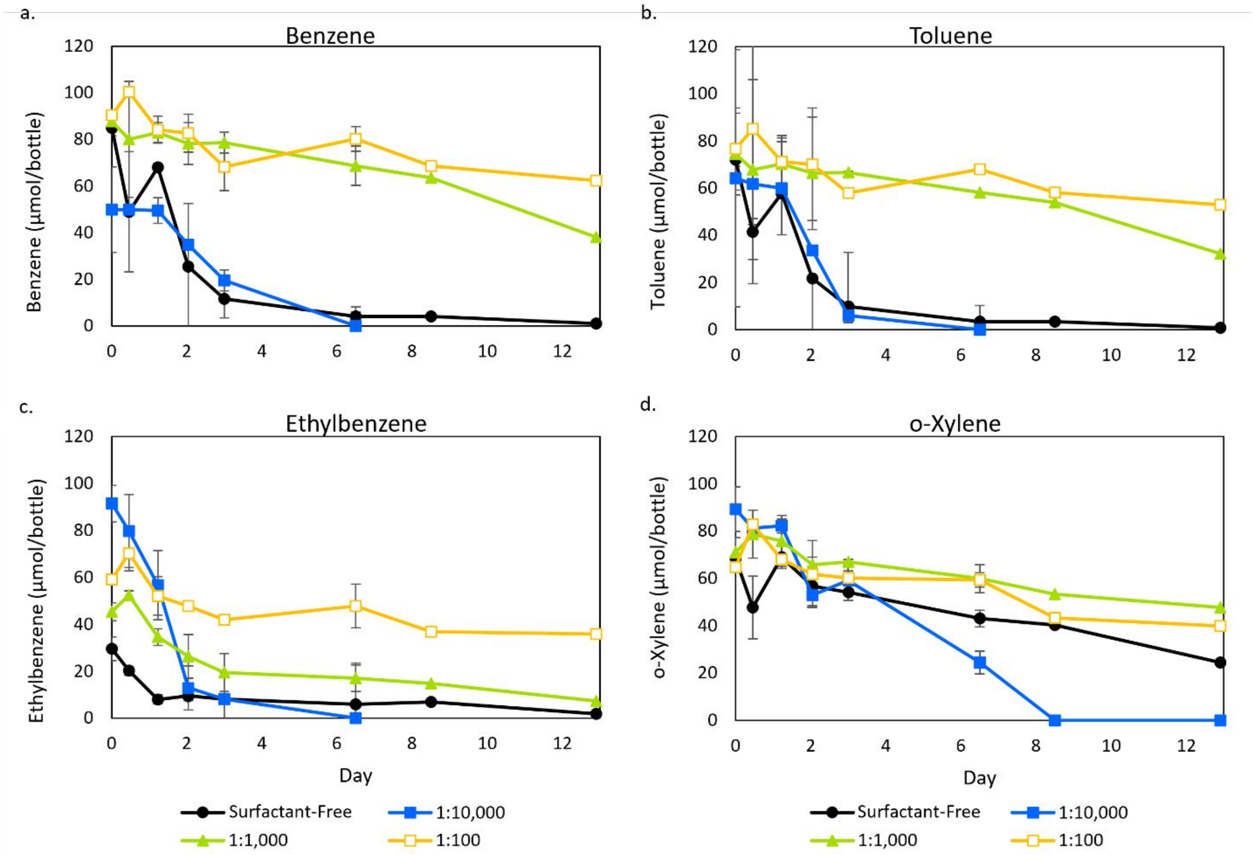

Figure S3. Aerobic BTEX degrading culture consuming a) benzene, b) toluene, c) ethylbenzene, and d) ortho-xylene in the presence of dilutions of AFFF. Error bars represent standard deviation of triplicate cultures.

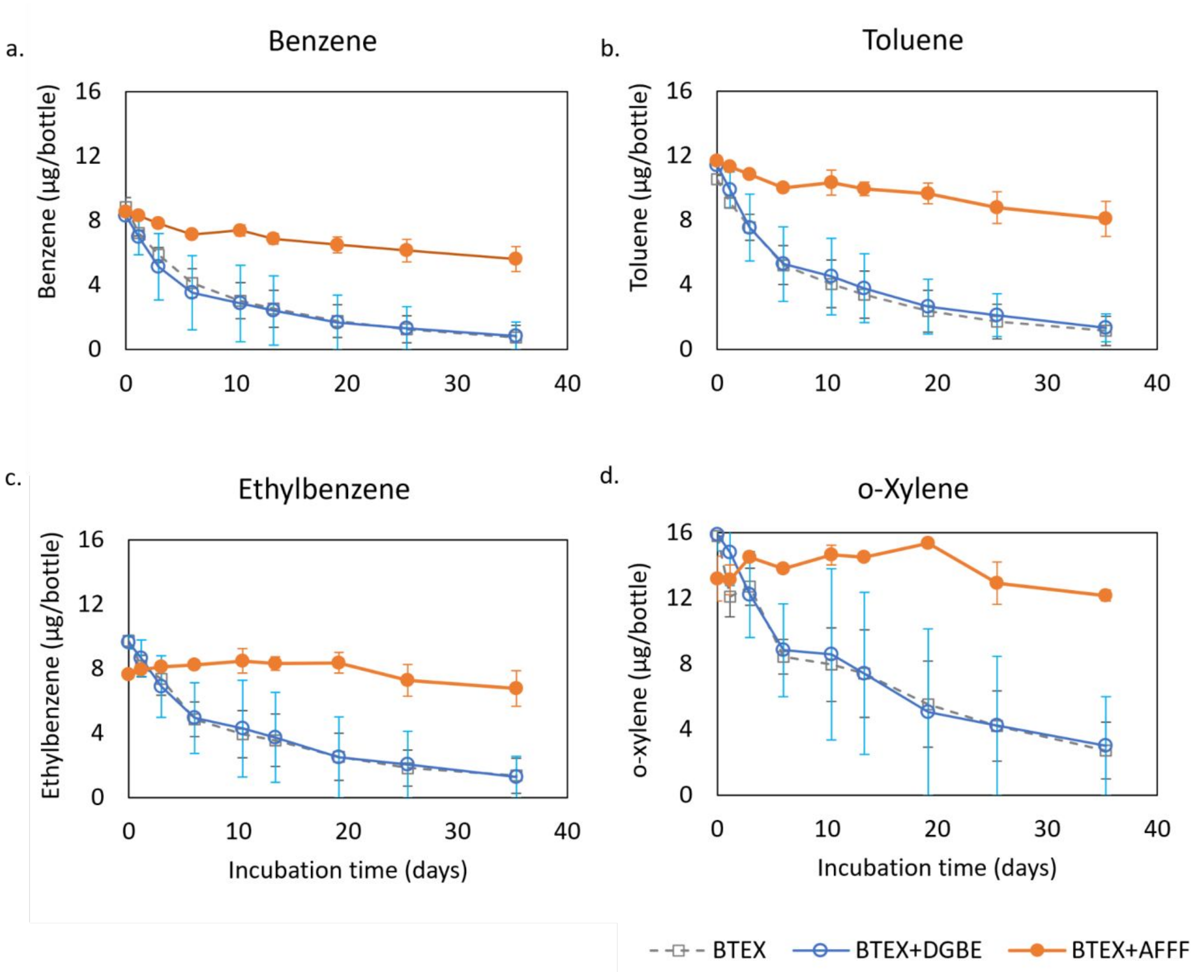

Figure S4. Aerobic BTEX biodegradation inhibition separating DGBE vs. all AFFF components combined. No inhibition was observed when DGBE was dosed in the same amount present in 1:1000 AFFF dilution, however stalling of BTEX biodegradation was observed when all components of AFFF were present. Error bars represent standard deviation of triplicate cultures.

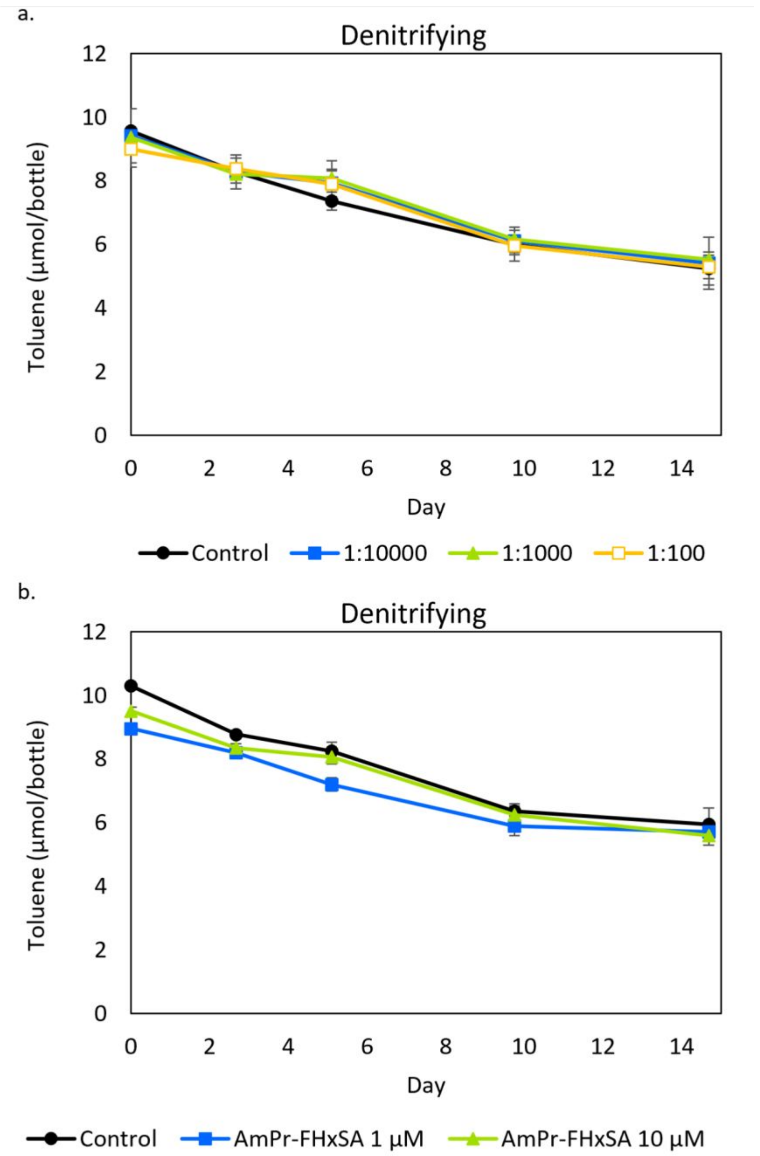

Figure S5. Anaerobic toluene degradation with the anaerobic enrichment culture under nitrate reducing conditions, exposed to a) dilutions of AFFF or b) two concentrations of AmPr-FHxSA. Error bars represent standard deviation of triplicate cultures.

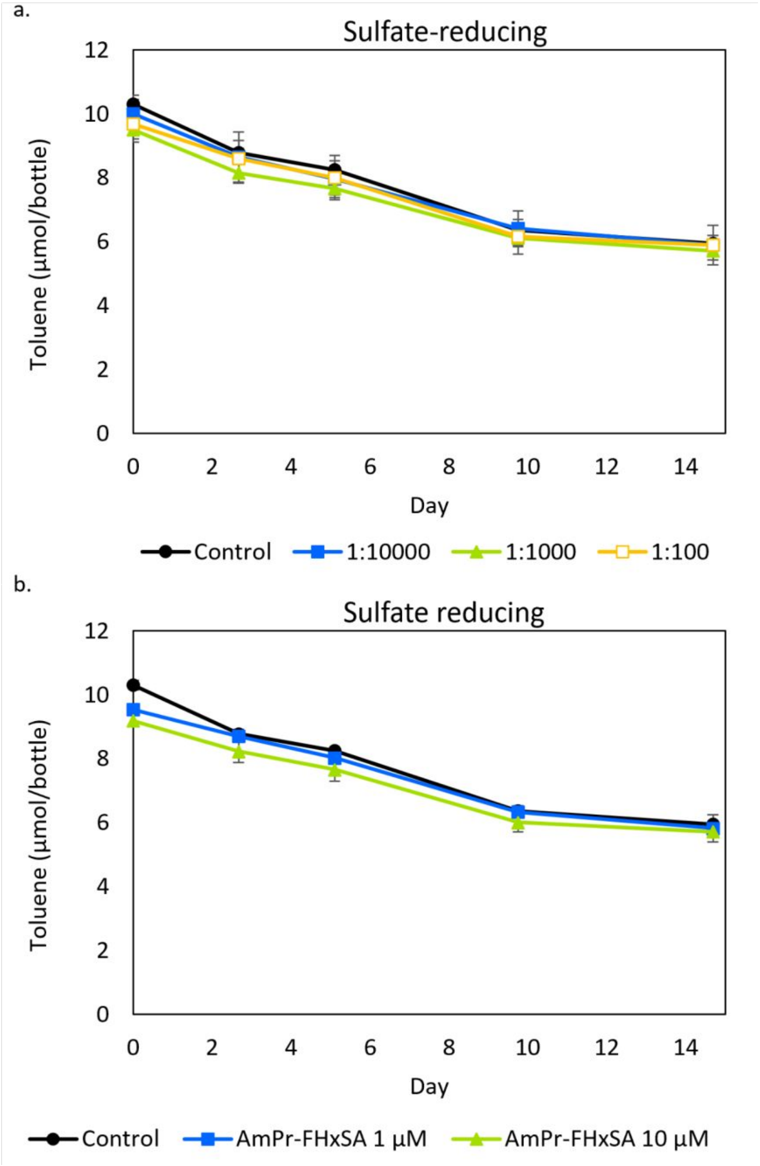

Figure S6. Anaerobic toluene degradation with the anaerobic enrichment culture under sulfate reducing conditions, exposed to a) dilutions of AFFF or b) two concentrations of AmPr-FHxSA. Error bars represent standard deviation of triplicate cultures.

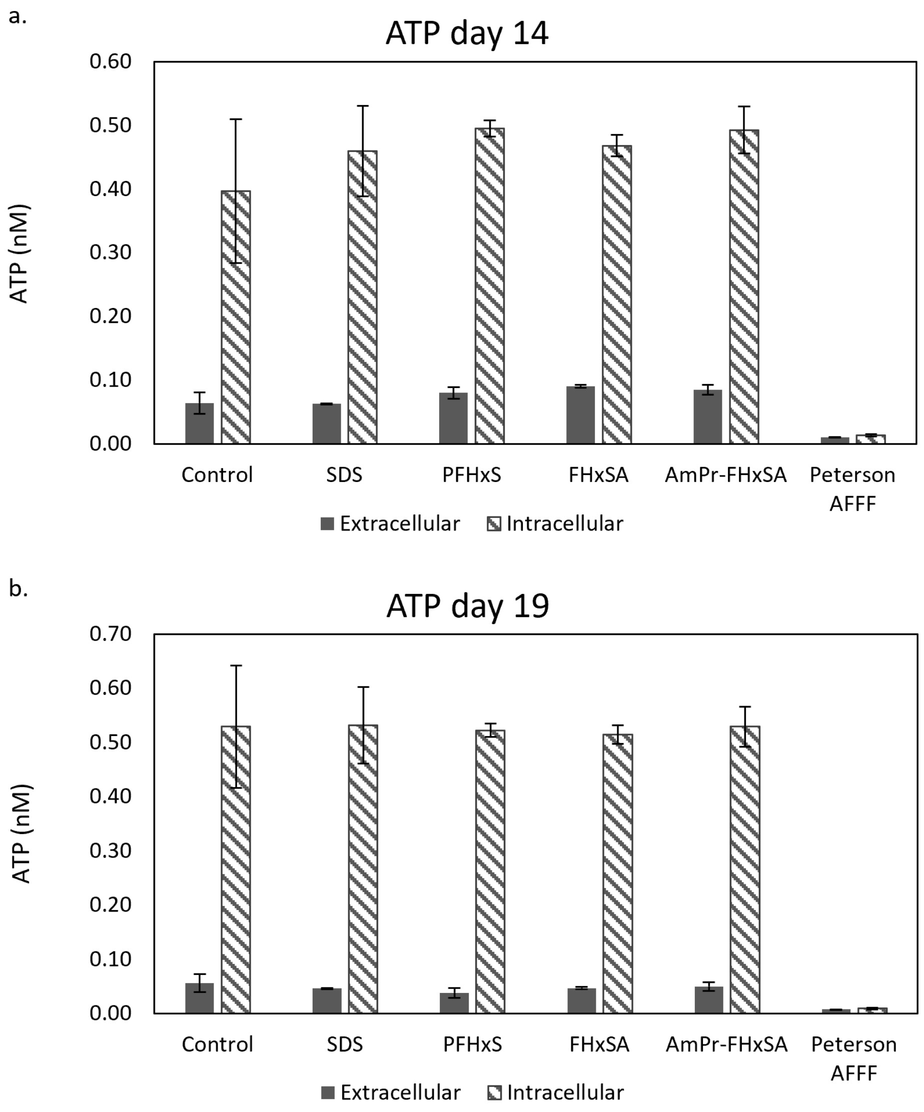

Figure S7. ATP concentrations in nM from the anaerobic TCE co-culture on day 14 of the 10 µM surfactant experiment, as shown in Figure S1e-h. Extracellular, shown in solid gray, represents ATP measured after filtering the biomass samples. Intracellular, shown in striped gray, represents ATP measured in total without filtering with the extracellular concentration subtracted. Error bars represent standard deviation of triplicate cultures.

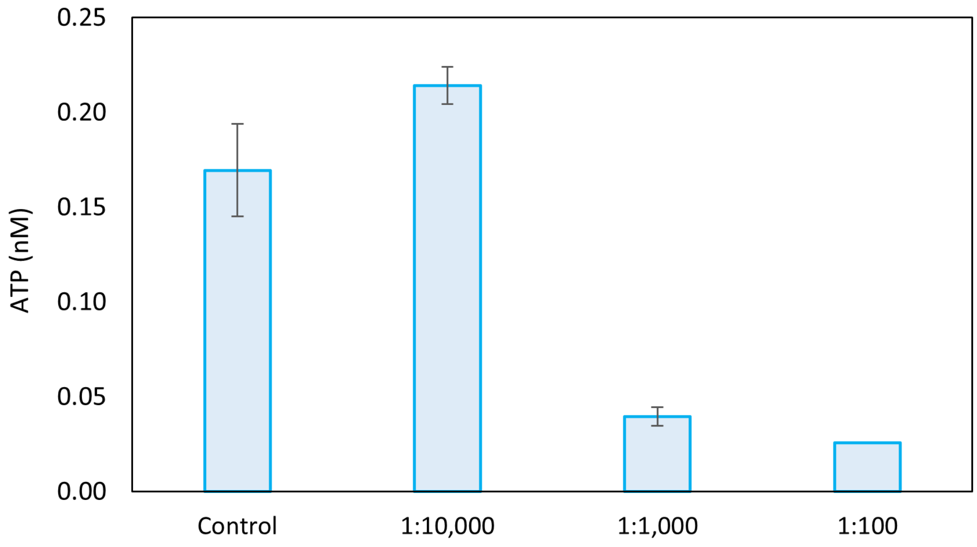

Figure S8. ATP concentrations in nM from the anaerobic TCE co-culture on day 16 of the AFFF dilution experiment as shown in Figure 2. Error bars represent standard deviation of triplicate cultures.

SI Tables

Table S1. Eluent gradient for LC-MS/MS metabolites method.

Time	0.1% formic acid and 10 mM ammonium formate (A)	95% acetonitrile, 5% water, 0.1% formic acid, with 10 mM ammonium formate (B)
0	0	100
2	0	100
7	30	70
7.7	30	70
9	60	40
9.5	60	40
10.25	70	30
12.75	0	100
17	0	100

Table S2. Analytes for metabolite LC-MS/MS analysis.

Compound	Retention Time (min)	Precursor Ion	Product Ion	Fragmentor	Collision Energy	Polarity
Glycine	12.75	76	30.1	25	5	+
Alanine	13.0	90.1	44.1	25	8	+
Sarcosine	12.9	90.1	44.2	46	12	+
Serine	12.65	106.1	60.1	25	8	+
Proline	13.0	116.1	70.1	50	16	+
Leucine/isoleucine	11.39	132.1	86.1	25	8	+
L-ornithine	13.79	133	116, 70.2	72	4, 16	+
Aspartic Acid	12.85	134	74	50	15	+
Lysine	12.75	147.1	84.1	50	16	+
Glutamic Acid	12.75	148.1	84.1	75	16	+
Methionine	11.40	150	104	75	8	+
Histidine	13.75	156.1	110	25	12	+
Phenylalanine	11.39	166.1	120.1	80	29	+
Arginine	13.79	175.1	70.1	75	28	+
L-citrulline	13.1	176	159.1, 70.2	72	4, 20	+
Tyrosine	11.35	182.1	136.1	25	12	+
m/z 393 unknown	2.7	393.3	57.2	80	40	+

Compound	Precursor Ion	Product Ion	Fragmentor	Collision Energy	Polarity
Glutathione-s2	613.2	355	100	5	+
393-pos	393.3	57.2	80	40	+
Glutathion	308	308	92	0	+
Oxaloacetic acid-con	271	157	92	8	-
Oxaloacetic acid	271	113	92	12	-
Tyrosine	182.1	136.1	25	12	+
L-citrulline	176	159.1	72	4	+
L-citrulline-conf	176	70.2	72	20	+
Arginine	175.1	70.1	75	28	+
Aconitic acid	169	125.1	68	4	-
Phenylalanine	166.1	120.1	25	5	+
Hilidine	156.1	110	25	12	+
Methionine	150	104	75	8	+
Glutamic acid	148.1	84.1	75	16	+
Lysine	147.1	84.1	50	16	+
Aspartic acid	134	74	50	15	+
L-ornithine	133	116	72	4	+
L-ornithine	133	70.2	72	16	+
Leucine	132.1	86.1	25	8	+
Isoleucine	132.1	86.1	25	8	+
Threonine	120	74.1	25	10	+
Valine	118.1	72.1	25	8	+
Proline	116.1	70.1	50	16	+
2-methyl oxobutanoate	116	74	180	10	-
Maleic acid	115	71.2	46	8	-
Serine	106.1	60.1	25	8	+
Sarcosine	90.1	44.2	46	12	+
Alanine	90.1	44.1	25	8	+
Glycine	76	30.1	25	5	+
